# Effect of Smoking on Subgingival Microbiome in Chronic Periodontitis: A 16S rRNA Sequencing Study

**DOI:** 10.3390/dj14010010

**Published:** 2025-12-23

**Authors:** Jazia A. Alblowi

**Affiliations:** Periodontology Department, Faculty of Dentistry, King Abdulaziz University, Jeddah 21589, Saudi Arabia; jaalblowi@kau.edu.sa

**Keywords:** 16S rRNA, fusobacterium, ion torrent, microbiome, periodontitis, smoking

## Abstract

**Background**: Smoking has a detrimental effect on the periodontal condition. Smoking intensity has recently been considered as a criterion for grading periodontitis cases. However, the influence of smoking intensity on the subgingival microbial community has not been evaluated in depth. This cross-sectional analytical study aims to assess the differences in the subgingival microbiome in adult patients with chronic periodontitis and different smoking habits (heavy smokers versus moderate smokers versus non-smokers). **Methods**: Sixty patients diagnosed with chronic periodontitis were grouped according to their daily smoking intensity as follows: group I (smoke ≥ 10 cigarettes/day), group II (smoke < 10 cigarettes/day), and non-smokers (group III). For each patient, samples from subgingival plaque were harvested from the deepest three periodontal pockets, and their 16S rRNA was sequenced using the S5 Ion Torrent platform. Sequences were clustered in taxonomic units, and the microbial diversity was expressed using the Shannon index or Simpson index, while the abundance of the microbial species was expressed using the Chao index. **Results**: Bacterial diversity was lowest in the heavy smoker group (group I) and highest in non-smokers (group III). *Veillonella*, *Streptococcus*, *Prevotella*, *Fusobacterium*, and *Dialister* were found to have different prevalences in the three study groups. *Campylobacter* decreased and *Fusobacterium* increased as a function of the number of cigarettes smoked per day. The moderate smoker group showed a higher abundance of *Spirochaetes.* At the species level, the heavy smoker group (group I) showed a higher abundance of *Fusobacterium* compared to the other two groups. **Conclusions**: Greater smoking intensity has been associated with higher *Fusobacterium* abundance, together with decreased diversity of the subgingival microbiome, establishing a more stable putative subgingival bacterial environment.

## 1. Introduction

Periodontitis, the sixth most common disease globally, is one of the main public health issues, significantly contributing to dental treatment costs [1,2]. Its negative sequelae include tooth loss, masticatory dysfunction, and esthetic deficit [2], and if left untreated, it can provoke or aggravate cardiovascular diseases, diabetes mellitus, and pulmonary diseases [3].

The primary etiological factor of periodontitis is a shift in the oral bacterial community from equilibrium. This dysbiosis can either directly damage periodontal structures or stimulate host inflammatory responses that mediate damage [4].

Besides dysbiosis, other factors play an important role in modifying the speed of progression and the pattern of teeth affected by periodontitis. Among these, tobacco smoking (recently considered as nicotine dependence and a chronic relapsing medical disorder rather than a habit) constitutes a major modifying factor [5]. It increases the risk of periodontitis by about two- to five-fold [5] and accounts for about 42% of periodontitis cases [1].

Due to its detrimental effect on the periodontal apparatus, smoking was recently established as a criterion for grading and diagnosing periodontitis cases [5].

Periodontitis is graded into three categories according to tobacco smoking: non-smokers, smokers who consume less than ten cigarettes per day, and smokers who consume ten or more cigarettes per day. Patients who are graded in the last category are expected to develop a more severe stage of periodontitis in a shorter period and respond poorly to standard treatment [6].

However, grading alone does not provide sufficient information about the periodontal disease process. Omics approaches, including genomics, provide an understanding of oral microbial communities [7].

In addition to using clinical grading data, Papapanou et al. (2018) [2] recommended the identification of genetic and microbial markers that can elucidate the differences between forms of periodontitis.

Accordingly, investigating the subgingival microbiome could be considered a reliable method to evaluate the impact of treatment on targeting pathologic changes [8].

Using such methodology to understand the differences between smoking categories can revolutionize clinical diagnostics [9] and help explain the impact of smoking on the oral microbiome and the influence of each smoker category on treatment. This can help in providing further insights into the pathogenesis of periodontitis and personalized dental treatment and prophylaxis [9].

Current research is concerned with detecting the differences in periodontal health and microbial makeup between smokers and non-smokers, thus providing an insight into the association of change in bacterial microbiome with the tobacco smoking intensity. Therefore, we performed this study to evaluate the differences in the oral microbiome of patients with periodontitis who are non-smokers versus moderate smokers versus heavy smokers using 16S rRNA sequencing. The null hypothesis of the study assumed that there would be no significant difference in microbial species diversity between the three groups.

## 2. Methods

### 2.1. Study Design

A cross-sectional analytical study was conducted where samples from subgingival plaque were harvested from periodontal pockets in smokers with periodontitis with different smoking intensities, and 16S rRNA was sequenced after purification and isolation of bacterial genomic DNA.

### 2.2. Study Setting

This study was carried out in the outpatient clinic of the Periodontology Department, Faculty of Dentistry, King Abdulaziz University, Jeddah, Saudi Arabia. The research was approved by the Research Ethics Committee at the faculty prior to research commencement (Approval No. 127-10-18; Approval Date: 14 January 2023). Additionally, this study was registered at ClinicalTrials.gov (Identifier No. NCT05995158).

Sixty smoker patients were recruited and grouped according to their smoking intensity as follows: group I (smoke ≥ 10 cigarettes/day), group II (smoke < 10 cigarettes/day), and non-smokers (group III). They also met the eligibility criteria regarding their periodontal condition. These criteria were (i) patients ≥18 years old, (ii) patients clinically diagnosed as having chronic periodontitis, (iii) patients who had undergone probing with a pocket depth (PD) ≥ 4 mm, and (iv) patients with clinical attachment loss (CAL) ≥ 3 mm at 2 or more non-adjacent teeth. Patients were excluded from the study if they had any systemic diseases, took any antibiotics in the past month, were undergoing radiotherapy, or were intellectually incapacitated.

### 2.3. Sample Size Calculation

Based on the power analysis conducted using G*Power 3.1.9.7, an a priori sample size estimation was performed for an independent samples *t*-test to detect the difference between group means. The analysis was two-tailed with a significance level (α) of 0.05 and a desired power (1 − β) of 0.80. The expected effect size (Cohen’s d) was calculated to be 0.97, which represents a large effect according to Cohen’s (1988) conventions [10].

This effect size was derived from group means of 6.7 and 7.1, with standard deviations of 0.3 and 0.5 for the studied groups. Under these parameters, the total required sample size was determined to be 60 participants, with 20 individuals in each group assuming equal allocation. The analysis yielded an actual power of 0.85, indicating a high probability of correctly rejecting the null hypothesis if a true effect exists [11].

### 2.4. Sampling Procedure

All patients signed an informed consent form before participation. Each patient’s full history of periodontal disease and smoking habits was obtained. Clinical periodontal examination and probing were performed, and CAL, bleeding on probing (BOP), and PPD were recorded.

The patients were categorized into three groups based on their smoking habits: group I, which included 20 periodontitis patients smoking ≥ 10 cigarettes/day; group II, which included 20 periodontitis patients smoking < 10 cigarettes/day; and group III, which included 20 non-smoking periodontitis patients.

For each patient, the three deepest periodontal pockets were selected for sampling, and cotton rolls were used to dry and isolate teeth in each site after gently removing and sampling supragingival plaque using sterile curettes (Hu-Friedy, Inc., Chicago, IL, USA). The sampled plaque was swabbed with an OMNIgene^TM^ oral sampling kit for plaque and gums (OMR-110) (DNA Genotek, Ottawa, ON, Canada).

Using the OMNIgene^TM^ oral kit, the pooled samples of each patient were immediately transferred and suspended in stabilizing solution at room temperature according to the manufacturer’s instructions.

### 2.5. DNA Extraction

PrepMan Ultrasample preparation reagent was purchased from Life Technologies (Thermo Fisher, Carlsbad, CA, USA) and used to isolate and purify the bacterial genomic DNA in line with the manufacturer’s instructions.

### 2.6. Library Generation

Polymerase chain reaction (PCR) was used to amplify the 16S rRNA hypervariable regions V2-4-8 and V3-6, 7-9. Primer sets of the Ion 16S™ Metagenomics Kit purchased from Thermo Fisher (Waltham, MA, USA) were used. When combined, the two primer pools can identify a range of bacteria. Two reactions were performed, including one for each primer. Agencourt AMPure XP beads purchased from Beckman Coulter and 70% ethanol were then employed to purify PCR amplification products based on the manufacturer’s instructions.

A Qubit™ dsDNA HS Assay Kit (Life Technologies, Thermo Fisher, Carlsbad, CA, USA) was utilized to analyze the DNA input for amplicon library preparation, and the Ion Plus Fragment Library Kit was used (Life Technologies, Thermo Fisher, Carlsbad, CA, USA) to purify the pooled amplicons following the manufacturer’s instructions.

### 2.7. Metagenomic Sequencing

Bacterial genomic DNA was isolated from from the samples and purified using PrepMan Ultra Sample Preparation Reagent (Thermo Fisher Scientific, Carlsbad, CA, USA) according to the manufacturer’s instructions.

To prepare for Ion OneTouch reactions, quantitative PCR was conducted to quantify the concentration of purified amplicons. The Ion 520 and Ion 530 Kits from Life Technologies (Thermo Fisher, Carlsbad, CA, USA) and the Ion OneTouch 2 system purchased from Thermo Fisher (Waltham, MA, USA) were used to amplify and enrich the amplicons. The enriched amplicons were loaded onto Ion 318™ Chip Kit v2 (Thermo Fisher Scientific, Carlsbad, CA, USA) and were sequenced using an Ion S5 system (Thermo Fisher Scientific, Waltham, MA, USA).

### 2.8. Sequence Analysis

The results were evaluated utilizing an Ion Reporter™ Ion 16S™ Metagenomics Kit and an analysis module (https://ionreporter.thermofisher.com/ir/, accessed on 15 September 2025). Alpha diversity values were calculated at 17,291 reads as the smallest set of sequences. Sequences were then clustered in taxonomic units. The microbial diversity in the samples was expressed using the Shannon index or Simpson index, while the abundance of the microbial species was expressed using the Chao index. The samples were tested versus the *E. coli* DH10B Ion Control Library. Operational taxonomic units (OTUs) were identified and clustered based on the Greengenes (version 13.8) 16S rRNA gene database, using a 97% sequence similarity threshold (McDonald et al., 2012). Subsequently, OTU filtering was performed by removing chimeric sequences, excluding singleton OTUs detected fewer than three times, and discarding OTUs with a total observation count of less than 100 [12]. Negative controls consisting of blank specimens were processed with each batch; no amplification products or microbial sequences were detected, confirming an absence of contaminant DNA. The absence of amplification and microbial signal in the blanks indicated that no reagent-derived or laboratory contamination (“kitome”) influenced downstream analyses.

The microbial diversity in the samples was expressed using the Shannon index or Simpson index, while the abundance of the microbial species was expressed using the Chao index. Blinding was carried out on the part of the analyst of the samples, who was unaware of the participants belonging to any of the study groups.

### 2.9. Statistical Analysis

Baseline and clinical data were presented as mean and standard deviation values. For numerical data, the analysis of variance (ANOVA) was used for group comparisons. Further analysis with Kruskal–Wallis was provided as a more appropriate test for violation of normality (File S1) [13].

Sequencing was carried out using the Ion S5 system sequencer. The reads were classified using the 16S Metagenomics Analysis module in the Ion Reporter Software. The relative taxon prevalence and abundance in the groups were described, and taxonomic summaries at all levels, from the phylum to genus, were determined and plotted into charts using Krona tools (GitHub, Inc., San Francisco, CA, USA). The software provided information up to the species level; however, not all data could be visualized when species were presented in our plots due to the large number of species and the small size of the chart. Therefore, we only included plots up to the genus level. Alpha diversity measures were computed using the Ion Reporter Software. The study groups were compared based on diversity, richness, and evenness indices (ANOVA, *p* < 0.05). Version 20 of the IBM SPSS program for Windows was used to analyze all data where a significance level of *p* ≤ 0.05 was determined.

## 3. Results

In this study we recruited 60 adult smoker patients diagnosed with chronic periodontitis. The patients were grouped as follows based on their smoking habits: heavy smokers consuming 10 or more cigarettes/day (group I), moderate smokers consuming 10 cigarettes/day (group II) or less, and non-smokers (group III).

### 3.1. Demographic and Clinical Parameters

The participants did not differ significantly in demographic characteristics since the test and controls were previously matched by age and gender. The participants’ ages ranged from 24 to 70 years, with a mean age of 39.2 years. Likewise, the groups did not differ in clinical characteristics, but group II had a lower percentage of BOP sites (Table 1).

Regarding collection sites, the mean PPD was 6.25 mm (±1.1) in group I, 5.7 (±0.7) in group II, and 5.5 (±0.9) in group III. On average, clinical attachment loss was observed in 46.7% (±10.2) of sites in group I, 36.6% (±12.5) in group II, and 26.7% (±8.8) in group III. For group I, 10 patients had stage III, grade C periodontitis and the other 10 had stage IV, grade C. Group II had 12 patients with stage III, grade B periodontitis and 8 with stage IV, grade B. In the control group, 14 patients had stage III, grade A periodontitis and 6 with stage IV, grade A.

### 3.2. Characteristics of 16S rRNA Gene at Phylum/Genus/Species Levels

The three study groups generally showed an abundance of four major phyla: *Firmicutes*, *Bacteroidetes*, *Proteobacteria*, and *Fusobacteria*. The non-smoker group showed a significantly higher percentage of *Firmicutes* compared to the two exposure groups. *Fusobacterium* abundance increased gradually from low levels in non-smokers (group III) to relatively higher levels in moderate smokers (group II) and to the highest levels in heavy smokers (group I). Furthermore, moderate smokers (group II) showed a higher abundance of *Spirochaetes* compared to groups I and III.

**Figure 1:**
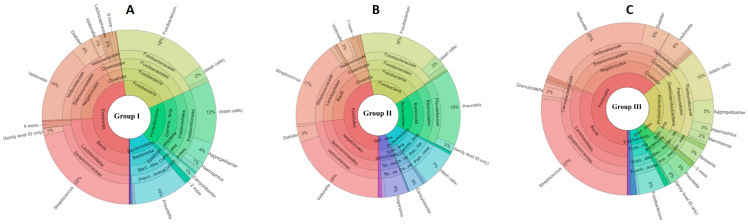
Representation of abundant operational taxonomic units (OTUs) in the three study groups. The colors in Figure 1 are used solely as visual identifiers for different bacterial taxa and do not represent biological or clinical states. (**A**) Group I: Subgingival microbial profile characterized by a higher relative abundance of periodontal-associated taxa and reduced microbial diversity. (**B**) Group II: Intermediate microbial composition showing partial shifts in taxonomic distribution compared with Group I and Group III. (**C**) Group III: Subgingival microbiome with a more balanced taxonomic distribution and greater microbial diversity.

At the genus level, the most abundant genera in the three groups were *Veillonella*, *Streptococcus*, *Prevotella*, *Fusobacterium*, and *Dialister*. In the moderate smoker group, *Pasteurella* and *Treponema* were observed, while the non-smoker group showed higher abundances of *Campylobacter*, *Porphyromonas*, and *Aggregatibacter* than the exposure groups. The abundance of *Campylobacter* decreased and that of *Fusobacterium* increased with a greater number of smoked cigarettes per day (Figure 1 and File S1).

At the species level, the heavy smoker group (group I) showed high abundances of *Fusobacterium nucleatum*, *Veillonella alcalescens*, and *Prevotella oris*. The moderate smoker group (group II) shared high abundances of *Veillonella alkalescens* and *Prevotella oris* with group I, followed by *Dialister pneumosintes* and *Veillonella rogasae*. Lastly, the non-smoker group exhibited significantly higher percentages of all species of *Veillonella*: *V. dispar*, *V. alcalescens*, *V. atypica*, *V. rogosae*, and *V. parvula*.

Overlaps among the results of the three study groups were observed. While group II somehow became distinguishable from the control group, group I results gradually appeared more dispersed from those of the control group.

### 3.3. Species Richness and Refraction Curves

Different indices were used to evaluate the samples’ diversity, richness, and evenness, specifically representing the degree of diversity of organisms for each group at the phylum, genus, and species levels.

**Figure 2:**
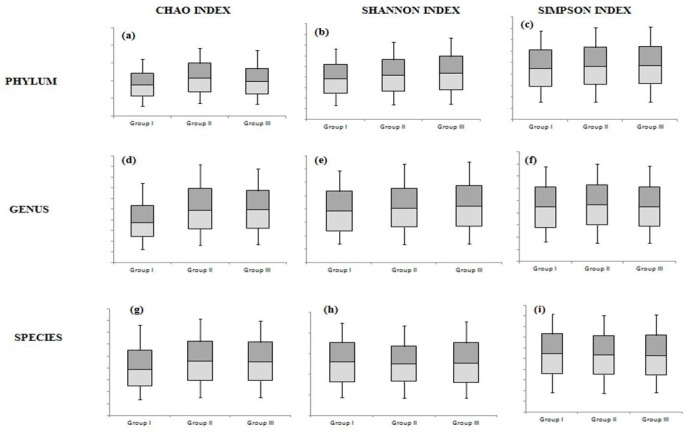
The differences among the 3 study groups at the phylum level according to the Chao index (**a**), Shannon index (**b**), and Simpson index (**c**); at the genus level according to the Chao index (**d**), Shannon index (**e**), and Simpson index (**f**); and at the species level according to the Chao index (**g**), Shannon index (**h**), and Simpson index (**i**).

At the phylum and genus levels, the greatest diversity was detected in the non-smoker group, and the lowest was observed in the heavy smoker group (group I). However, these differences were statistically insignificant (Figure 2 and File S1).

## 4. Discussion

The diagnosis of periodontitis has recently been graded based on several criteria, including the intensity of tobacco smoking [6]. In this study, we tested the hypothesis that the subgingival plaque microbiomes of patients with chronic periodontitis may differ based on smoking intensity. To explore this, we recruited patients with chronic periodontitis and different smoking habits. Subgingival plaque samples were harvested, and their 16S rRNA was sequenced using the S5 Ion Torrent platform.

The present study included three groups of patients with no significant differences in their baseline demographic characteristics. The smoking habits of the sampled population favor males over females [14]; therefore, the study only included males.

The differences were not statistically significant, as tested by the Kruskal–Wallis statistic. Accordingly, the null hypothesis of the study is accepted. The non-significance of the results could be attributed to sample variation or sequencing depth.

Clinically, the CAL and probing depth indices worsened with increasing smoking intensity. This is consistent with the direct correlation reported between tobacco smoking and the extent of probing depth and attachment loss [15]. Smoking has also been suggested to create a commensal-poor, pathogen-rich microenvironment, which renders the microbiome “at risk for harm” [16], and it was further found to change bacterial colonization in favor of disease-associated pathogens [17]. Moreover, the moderate smoker group had a lower BOP index compared to the other two groups. Such a finding can be due to the fact that certain factors can mask gingival bleeding, like increased fibrosis with long-standing chronic inflammation. In addition, these differences were statistically insignificant.

Tobacco smoking may directly and indirectly promote periodontal disease [18]. The toxins in smoke may alter the microbial community directly through oxygen deprivation and an antibiotic effect, causing the overgrowth of pathogenic anaerobes [19]. Tobacco smoking also has a detrimental impact on immunity, the metabolome, and DNA replication and repair [20]. Together, all of these mechanisms aggravate periodontal disease [18].

However, evidence of the variation in subgingival bacterial community between non-smokers and smokers is largely controversial [21].

Subgingival plaque samples were analyzed utilizing various methods. Previously, targeted selective approaches detecting certain microorganisms have been implemented [22], and some studies found no difference in smoker versus non-smoker subgingival bacteria [23,24,25], while others detected increased levels of periodontitis-associated pathogens in smokers [26,27,28].

With the advent of metagenomics, open-ended techniques have been used to sequence the nucleic acids of all microorganisms in a sample, thus detecting all the species in it [22]. A previous study found that selective and open-ended techniques detected remarkably different patterns of pathogens in the same sample at the species level and, to a lesser extent, at the genus level [22].

In the current study, we used the Ion S5 Ion Torrent platform, which utilizes primers for seven out of the nine hypervariable regions for the 16S rDNA in bacteria and is superior to other platforms that detect fewer hypervariable regions. This allows more diverse bacterial populations to be identified in the sequencing process. The platform depends on natural chemistry mechanisms by detecting hydrogen as a by-product of each newly formed phospho-diester bond, a technique that is much less sophisticated than those used in other platforms [29].

We observed the abundance of *Veillonella*, *Streptococcus*, *Prevotella*, *Fusobacterium*, and *Dialister* in the three study groups. Furthermore, we detected a decrease in diversity among bacterial phyla and genera with an increase in smoking intensity. Although the heavy smoker group showed the lowest diversity, the group differences were statistically insignificant.

Although the findings of this study were not the only to report statistically insignificant differences, this does not affect the reality of the registered changes driven by smoking. Our results corroborated the findings of Yu et al., who also detected the highest abundance of *Veillonella*, *Streptococcus*, *Prevotella*, and *Fusobacterium* genera in the subgingival samples of smokers and non-smokers. They recruited 23 smokers and 20 non-smokers from whom they harvested multiple samples, including subgingival plaque. They used the Illumina MiSeq platform that targeted the V3–V4 region of the 16S rRNA of the microbiome to sequence samples [30]. Their study’s findings concur with our results concerning the diversity of microbes, as they reported no significant difference among groups in the alpha diversity or taxa-relative abundance of microbial composition [30].

Two other studies detected no significant statistical differences in microbial diversity between smokers and non-smokers [19,22]. The first study was performed in the Netherlands with 30 patients who had periodontitis (15 non-smokers and 15 smokers), where subgingival plaque samples underwent pyrosequencing [22]. The second study, performed in Italy, analyzed the subgingival plaque samples of 12 patients who had periodontitis (six smokers and six non-smokers) and eight controls. The samples were sequenced using the Illumina MiSeq platform [19]. Both studies reported a higher abundance of some genera among smoker samples, including *Fusobacterium*, *Paludibacter*, and *Desulfobubus*.

In concordance with our results, one study performed in India reported higher diversity in non-smokers [20], contrary to another study conducted in Korea, which reported higher diversity in smokers and similar richness and evenness across smokers and non-smokers [31].

The former study compared samples from patients with chronic periodontitis. The researchers harvested granulation tissue from periodontal flap surgeries performed on five smokers and five non-smokers. The samples were sequenced using PacBio sequencing [19], while another study performed pyrosequencing on samples obtained from 134 smokers and 134 non-smokers [31].

Besides the genera detected in our study, the Indian study detected the dominance of *Streptobacillus*, *Streptococcus*, *Enhydrobacter*, and *Staphylococcus* in non-smokers [20]. The Korean study concluded that *Fusobacterium*, *Veillonella*, *Fretibacterium*, *Streptococcus*, *Corynebacterium*, TM7, and *Filifactor* were dominant, while other genera, such as *Prevotella*, *Aggregatibacter*, *Campylobacter*, *Haemophilus*, *Veillonellaceae* GQ422718, and *Prevotellaceae*, were depleted [31].

Our results revealed that *Campylobacter* abundance decreased and *Fusobacterium* increased with a greater number of smoked cigarettes per day, which is in line with the results of the Korean study [31]. *Fusobacterium* was found to be more profuse in the heavy smoker group. Despite not being traditionally considered to be among the most putative periodontal pathogens, *Fusobacterium* was identified to bridge biofilm bacteria. Therefore, it is important in subgingival biofilm buildup in addition to its local immunosuppressive action. In periodontal diseases, *Fusobacterium* is considered one of the most abundant genera. Among all *Fusobacterium* species, *F. nucleatum* is one of the key determinants of the altered subgingival microbiome in smoking patients regardless of ethnicity [4,22,31]. Future studies involving confirmation by species-species qPCR are recommended. *Fusobacterium nucleatum* is reported to be active in the mechanism of progression of periodontal disease. It works as an oxygen-tolerant scaffold producing metabolites such as butyrate and promotes hydrogen sulfide pathway production, thus favoring the growth of Porphyromonas gingivalis and other organisms. It also facilitates periodontal breakdown and modulates host response, reducing pain signaling. These metabolite-mediated effects may explain how shifts in Fusobacterium abundance/diversity affect disease progression [32,33,34].

As clarified, the available research mainly compares smokers and non-smokers. However, our research also investigates the microbial composition of moderate smokers. A characteristic increase in the abundance of *Spirochaetes* (mainly *Treponema* species) was observed when compared to both non-smokers and heavy smokers.

The oxygen tension in the oral cavity is known to drop due to smoking, creating a favorable environment for periodontal pathogens in periodontal pockets [35]. *Spirochaetes* are proven to be extremely virulent, allowing for tissue penetration and destruction [36]. Therefore, pathogens associated with periodontal disease were detected, mainly *Treponema*.

In this study, moderate smokers showed a more virulent microbial makeup compared to both non-smokers and heavy smokers. This may be attributed to high levels of inflammation, as bacteria are opposed by more healthy inflammatory reactions in moderate smokers compared to heavy smokers. In heavy smokers, the effects of nicotine became more pronounced in the form of a much-reduced inflammatory response, together with vasoconstriction. Accordingly, a more established subgingival environment was observed [37].

Studies investigating the subgingival microbiome exhibit major differences regarding the ethnic characteristics of participants, sampling techniques, and sequencing platforms, but many report findings similar to those of the present work, where smokers were found to harbor different strains in some species compared to non-smokers, including higher abundances of *Fusobacterium* [11,19,22,30,31,38], *Streptococcus* [19,29,30], *Prevotella* [21,29], and *Veillonella* [29,30] among smokers. Regarding the clinical relevance of the current work, and based on the reduced diversity of the oral microbiome detected in the results and reported by other authors favoring more pathogenic strains (Bizzaro et al.) and indicating a more diverse ecosystem, promoting more tissue destruction; therefore, the periodontist has to consider the smoking intensity in his risk assessment and treatment plan, considering regular recall visits and periodontal therapy (Hanioka). It is also reported that smoking cessation regains microbial ecosystem equilibrium and treatment prognosis (systematic review and meta-analysis). Therefore provision of the appropriate preventive strategies should be planned for those patients [22,39,40].

Some authors suggested that the metagenomic approaches provides powerful platform for mining and engineering microbial communities, enabling the development of targeted therapeutic strategies through manipulation of the micobiome [41], and although metagenomic technologies allow for accurate microbiome detection and analysis, these methods are expensive and technique-sensitive. They can be used in research, but they have not yet been employed to diagnose and adapt personalized medicine techniques. A recent systematic review has reported that periodontal microbiota signatures could potentially be used for the assessment of the development of periodontal disease [42]. In line with this concept, this study was performed to explore the microbial changes in periodontitis in smokers at different smoking intensities.

Limitations in the present study include potential confounding factors like age (a wide age range was used) and lifestyle, as well as individual variations in smoking history details and the immunologic background. Moreover, the definition of disease progression using measures like probing depth can also have high intra-visit variability. The study also focused on those with fully developed periodontitis, but this can miss important early changes or risk factors. However, there were no significant differences in age between the three groups, and a potential confounder such as oral hygiene was controlled for by taking the samples from three deepest pockets of the participants, and this pooled sampling provided a wider scope of the microbiome of each participant. Further limitation may be attributed to the cross-sectional nature of the study, which provides statistical associations rather than true relations between the smoking status and the microbiome detected.

Regarding the generalizability of the results, differences in how and when samples are collected (e.g., storage, timing, frequency, and specific location) can critically affect the outcome and result in differences between studies. The microbiome is dynamic and can change over time; hence, a single snapshot may not capture the full picture. Disease progression, often used to group samples, can be hard to measure reliably. Furthermore, differences in laboratory and analysis methods in different studies can lead to different results, making generalization difficult.

## 5. Conclusions

Increased smoking intensity was associated with a decrease in microbiome diversity and a higher relative abundance of Fusobacterium in the subgingival microbiome of patients with chronic periodontitis. However, they were not statistically significant. Some genera were detected in higher abundance, including *Veillonella*, *Streptococcus*, *Prevotella*, *Fusobacterium*, and *Dialister*. Future metagenomic and metabolomic analyses are recommended to elucidate functional changes associated with smoking intensity.

## Figures and Tables

**Table 1:** Demographic characteristics and clinical data of the participants.

	Smoke ≥ 10 Cigarettes/Day (Group I)	Smoke < 10 Cigarettes/Day (Group II)	Non-Smokers (Control) (Group III)	*p*-Value
Age	39.5 (10.2)	40.3 (7.3)	41.4 (9.6)	0.73
Sex				
Males	20	20	20	
Female	0	0	0	
No. of cigarettes/day	31.7 (11.3)	6.3 (2.5)	0	
PPD (mm)	6.25 (1.1)	5.7 (0.7)	5.5 (±0.9)	0.22
%BOP	87.6 (17.3)	74.3 (20.3)	89.6 (11.2)	0.001 *
CAL (mm)	4.14 (1.8)	4.05 (1.7)	3.73 (1.7)	0.63

* Statistical significance was determined using a one-way ANOVA test. %BOP, percentage of tooth sites with bleeding on probing; CAL, clinical attachment loss; PPD, periodontal probing depth.

## Data Availability

The raw data supporting the conclusions of this article will be made available by the authors on request.

## Supplementary Materials

The following supporting information can be downloaded at: https://www.mdpi.com/article/10.3390/dj14010010/s1, File S1: The Bacterial differences between the 3 study groups.
