# Supplementary material for: Effect of Smoking on Subgingival Microbiome in Chronic Periodontitis: A 16S rRNA Sequencing Study
Source: Dent J (Basel). 2025 Dec 23;14(1):10. doi: 10.3390/dj14010010 (PMC12840093; doi:10.3390/dj14010010)
Supplement: Supplementary file 1 [file dentistry-14-00010-s001.zip › dentistry-3935852-supplementary.pdf]

APPENDIX A

The differences between the 3 study groups at the level of phylum, genus and species.

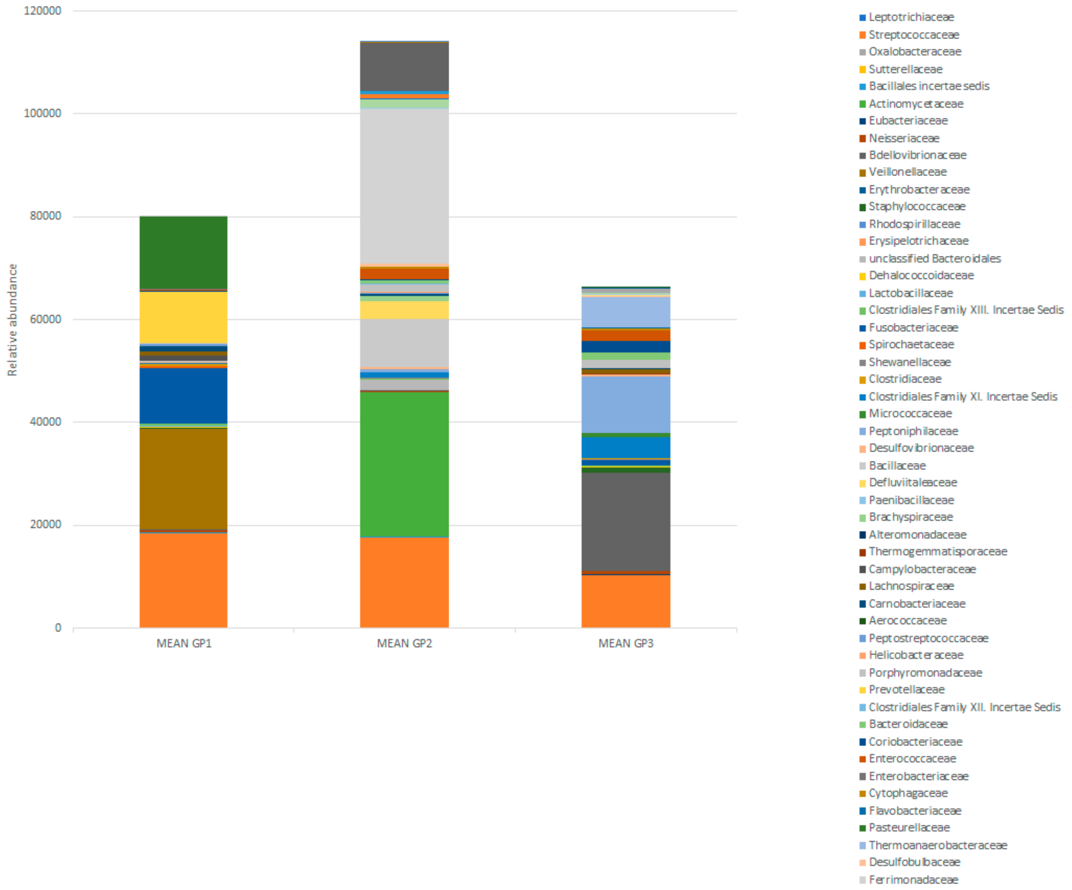

Figure 1 showing the difference between the 3 study groups at the level of phylum.

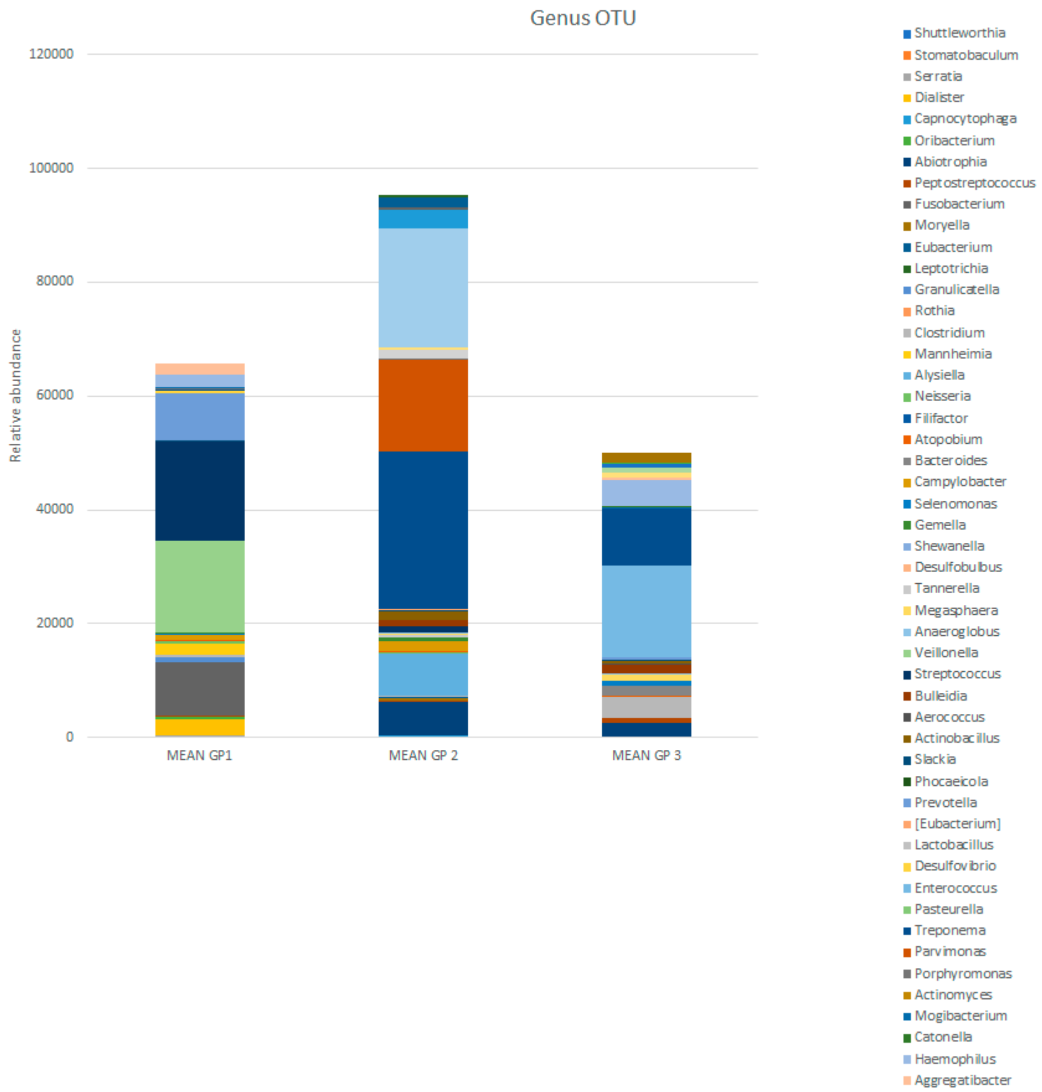

Figure 2 showing the difference between the 3 study groups at the level of genus.

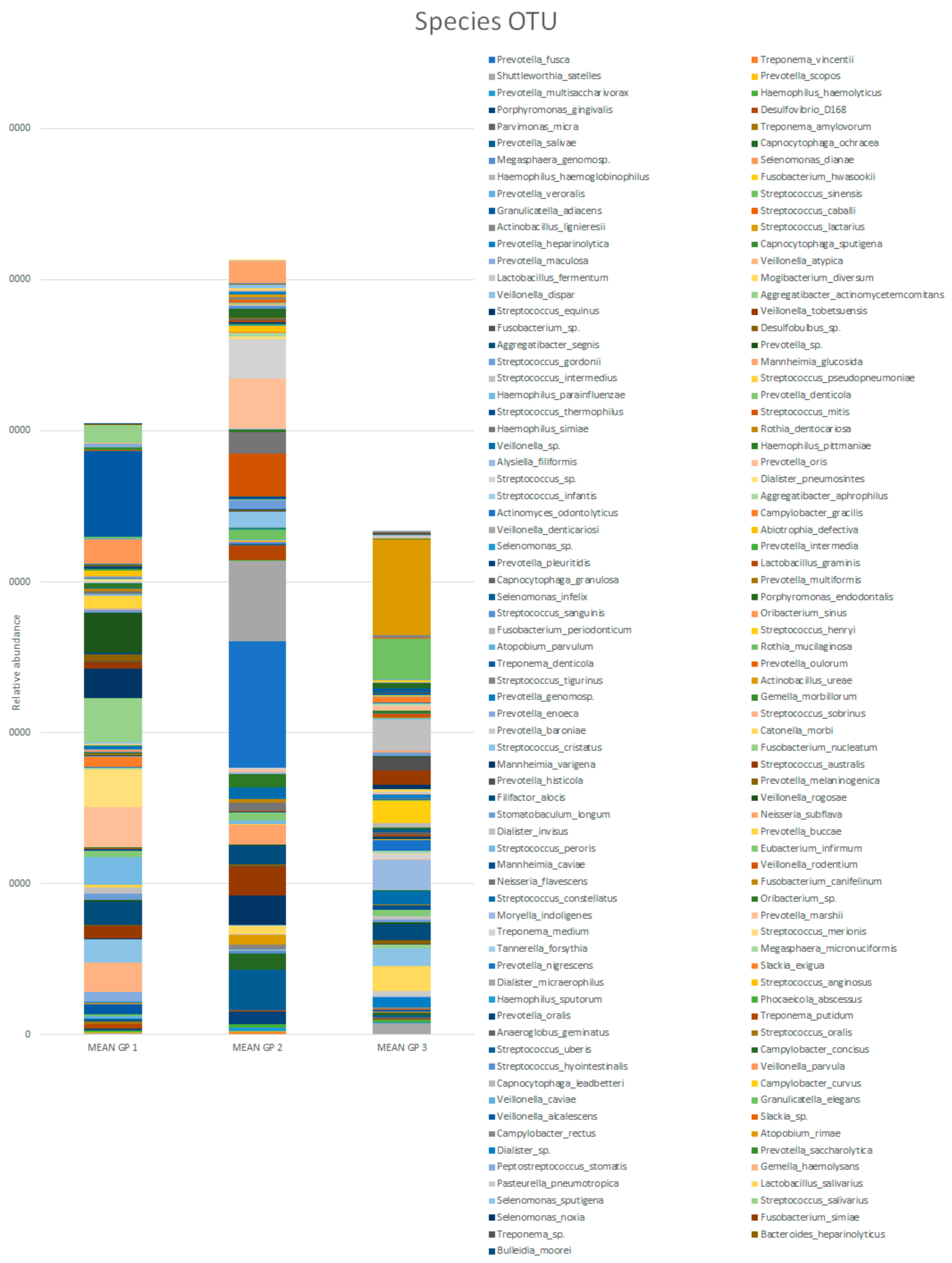

Figure 3 showing the difference between the 3 study groups at the level of species.

APPENDIX B

Alpha diversity in different groups at the phylum, genus and species levels:

	Smoking≥10 cigarettes/day (Exposure 1)	Smoker<10 cigarettes/day (Exposure 2)	Never smokers (Control)	*P* value
In phylum				
Chao	32.2 (5.02)	38.7 (4.15)	36.8 (7.83)	0.19
Shannon	2.65 (0.26)	2.92 (0.25)	3.08 (0.03)	0.05
Simpson	0.77 (0.06)	0.81 (0.03)	0.81 (0.03)	0.4
In genus				
Chao	29.8 (7.73)	36.7 (5.62)	35 (3.41)	0.14
Shannon	2.74 (0.49)	2.91 (0.34)	3 (0.31)	0.54
Simpson	0.77 (0.1)	0.8 (0.04)	0.78 (0.05)	0.85
In species				
Chao	76.4 (18.5)	81 (7.84)	79.5 (5.99)	0.78
Shannon	4.49 (0.38)	4.32 (0.29)	4.56 (0.53)	0.55
Simpson	0.92 (0.03)	0.9 (0.03)	0.91 (0.05)	0.77

*statistical significance was tested by one-way ANOVA test.

*statistical significance was tested by Kruskal-Wallis test.

	Shapiro-Wallis test p-value	test statistic **H**	effect size η2	*P* value
In phylum				
Chao	0.000226	**4.2664**	**0.11**	0.118
Shannon	0.000001176	**5.3505**	**0.16**	0.069
Simpson	1.067e-7	**0.8042**	**-0.057**	0.669
In genus				
Chao	0.0004282	**3.363**	**0.065**	0.186
Shannon	0.00001609	**0.7357**	**-0.06**	0.692
Simpson	4.689e-7	**0.3055**	**-0.081**	0.858
In species				
Chao	4.21e-7	**0.04964**	**-0.085**	0.975
Shannon	9.246e-7	**1.27**	**-0.035**	0.53
Simpson	4.753e-8	**1.1691**	**-0.04**	0.557
